# Second primary malignancy post immunotherapy: A case report of 2 cases

**DOI:** 10.1097/MD.0000000000037434

**Published:** 2024-03-08

**Authors:** Xian Miao, Shu Dong, Yuhua Tao, Xiaohui Yang, Shuijie Shen

**Affiliations:** a Department of Oncology, Nantong Hospital Affiliated to Nanjing University of Chinese Medicine, Nantong, China; b Department of Integrative Oncology, Fudan University Shanghai Cancer Center, Shanghai, China.

**Keywords:** immune checkpoint inhibitor, immunotherapy, nonsmall cell lung cancer, pancreatic cancer, renal cancer, second primary malignancy

## Abstract

**Rationale:**

Immune checkpoint inhibitors have shown high efficacies as the first-line treatment of various advanced malignancies. Yet, the effect and practice patterns of immune checkpoint inhibitors on the second primary tumors are still unclear. Second primary malignancy post immunotherapy, there is paucity in such cases being reported.

**Patient concerns:**

We report 2 cases of a 57-year-old woman with nonsmall cell lung cancer and a 69-year-old man with metastatic clear cell renal carcinoma treated with immunotherapy who developed second primary malignancies during the therapy.

**Diagnosis:**

Second primary malignancy during the therapy.

**Interventions:**

In addition to the treatments of the second primary malignancies, maintenance immunotherapy was continued for the patients.

**Outcomes:**

Overall survival in both patients was longer than 12 months, and the treatments were well tolerated. The adverse reactions mainly included depigmentation of hair and facial and limb skin in patient 1 and diarrhea in patient 2.

**Lessons:**

It is necessary to recognize that the second primary malignancy may occur during the immunotherapy, and more clinical studies and practices are still needed for the adjustment of the regimens of immunotherapy. Full diagnosis, timely treatment, and long-term regular follow-up have important significance for patients with malignancies.

## 1. Introduction

Implementation of immune checkpoint inhibitors (ICIs) has substantially improved the treatment effects in patients with advanced or metastatic nonsmall cell lung cancer (NSCLC).^[1]^ The application of the PD-1 pathway targeting ICI plus targeted-therapy has marked a new era of kidney cancer treatment.^[2]^ However, the improvement of immunotherapy regimen standardization and adverse effects related to immunotherapy need to be further explored. Also, the effect of ICIs on second primary tumors is still unclear.

Herein, we reported 2 cases treated with immunotherapy who developed second primary malignancies during the therapy, including clinical and imaging findings.

## 2. Case presentation

### 2.1. Case 1

A 57-year-old woman was admitted to our hospital in early 2020 after complaining about an irritable cough. The woman had a history of rectal cancer, and her sister and brother died of intestinal cancer. Computed tomography (CT) of the chest showed cancer in the superior lobe of the left lung; the left hilar vessel was involved, and a slight enlargement of mediastinal lymph nodes was found. Lung biopsy and pathological examination of the lesion in the superior lobe of the left lung indicated NSCLC. Immunohistochemistry showed TTF-2 (−), NapsinA (−), focal CK7 (+), P40 (+), P63 (+), CDX-2 (−), Villin (−), Her2 (−), P53 (+, 20%), Ki67 (+, 70%), EGFR (++), and P16 (+), which suggested a moderately differentiated squamous cell carcinoma. The next-generation sequencing (NGS) showed KRAS exon2 mutation (mutation rate 31.4%), while no other mutation was detected. As the tumor invaded great vessels, the multidisciplinary discussion suggested the following: Thoracic Surgery Department suggested that the risk of surgery was very high, and thus surgical treatment was not recommended; the Radiotherapy Department suggested reassessment after 2 cycles of chemotherapy; the Oncology Department suggested chemotherapy.

**Figure 1.**
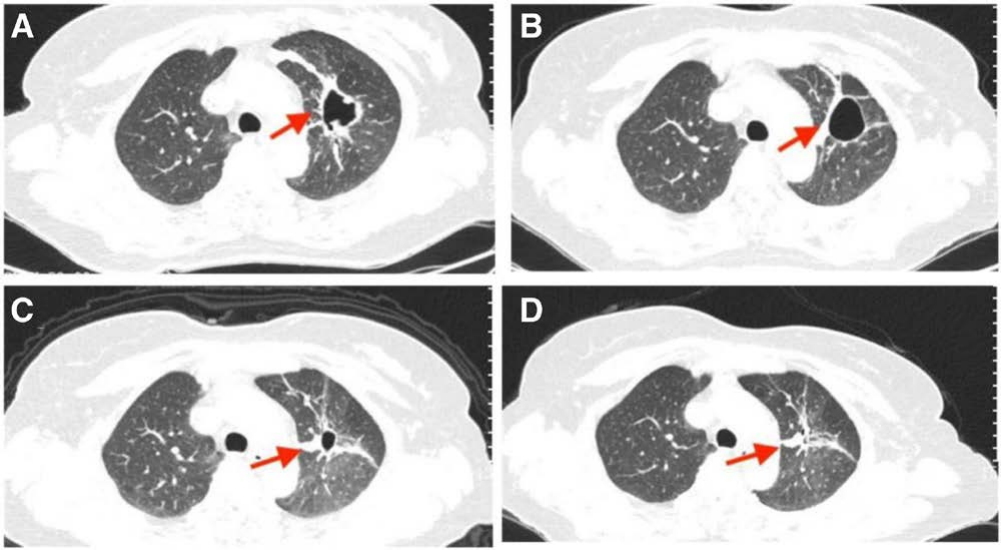
Patient 1: Chest CT images at a different stage. The major lesion (red arrow) is in the superior lobe of the right lung. (A) Before immunotherapy on June 17, 2020. (B) After 4 cycles of combination immunotherapy on October 5, 2020. (C) A pancreatic lesion was found on March 9, 2021, and chest CT scanning showed persistent remission of the pulmonary lesion. (D) Chest CT image on June 9, 2021 showed persistent remission of the pulmonary lesion. CT = computed tomography.

The patient received 2 cycles of chemotherapy (abraxane plus cis-platinum) starting on April 7, 2020, and the treatment efficacy assessment showed partial response (PR). Doctors from the Radiotherapy Department were then invited for consultation again, and they warned about the risk of bleeding during radiotherapy due to a cavity in the left lung; yet, the woman and her family refused radiotherapy. Instead, she started anti-PD-1 combination chemotherapy (anti-PD-1, abraxane, and cis-platinum) on June 23, 2020. After 4 cycles, the assessment showed that the treatment efficacy was PR (Fig. 1A and B). Afterward, anti-PD-1 monotherapy was given as maintenance therapy.

**Figure 2.**
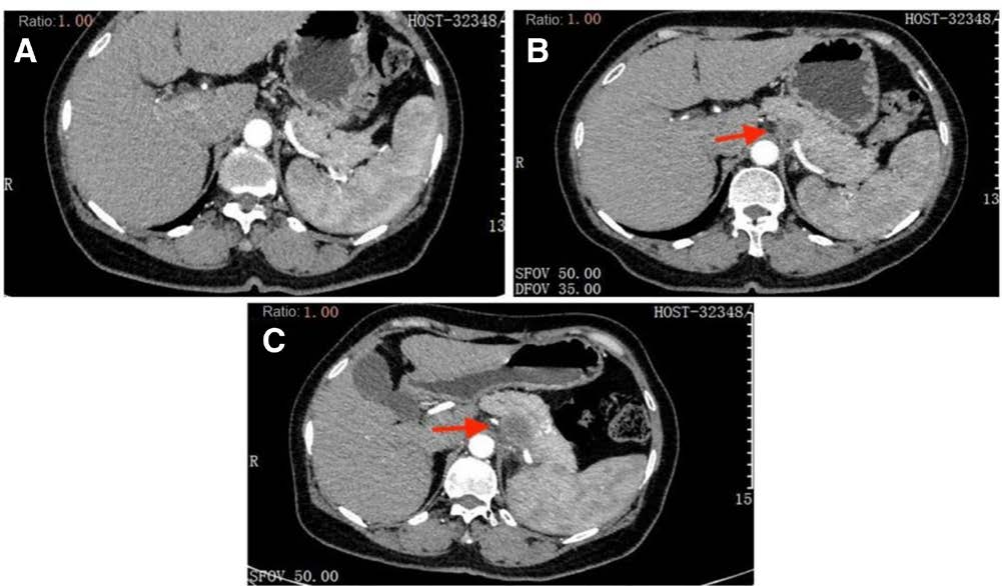
Abdominal CT images at different stages. The major lesion (red arrow) is at the pancreatic body. (A) Abdominal CT image on October 5, 2020 showed no space-occupying lesion in the pancreas. (B) Examinations showed persistent remission of the pulmonary lesion during immunotherapy, but the abdominal CT scanning on March 9, 2021 showed a space-occupying lesion in the pancreas. (C) CT scanning on June 9, 2021 showed persistent remission of the pulmonary lesion during the immunotherapy, but the pancreatic lesion enlarged. CT = computed tomography.

**Figure 3.**
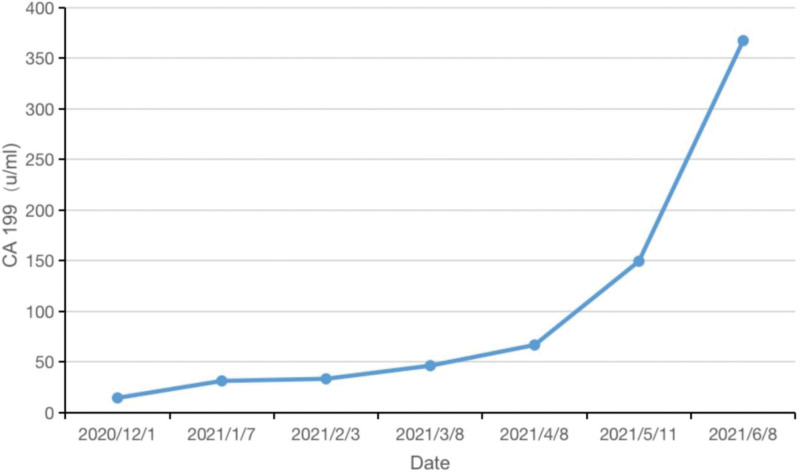
Changes of CA19-9 from December 1, 2020 to June 8, 2021.

Her serum CA19-9 level was within the normal range before January 2021, and multiple imaging examinations detected no extrapulmonary lesions (Fig. 2A). However, on January 7, 2021, her CA19-9 level was 31.16 µ/mL and continued to increase. On March 9, 2021, CT of the lungs showed a new pancreatic lesion (Fig. 2B). The woman refused further examinations of the pancreatic lesion. As the lesion in the lung was still in persistent remission (Fig. 1C and D), the anti-PD-1 therapy was continued, but CA19-9 increased to 367.5 µ/mL on June 9, 2021 (Fig. 3). A second CT scanning showed that the cavity in the superior lobe of the left lung shrank, a new metastatic tumor appeared in the liver, and the pancreatic lesion enlarged compared to that on March 9, 2021 (Fig. 2C); in addition, the pancreatic lesion enwrapped the splenic artery, indicating the involvement of splenic artery.

**Figure 4.**
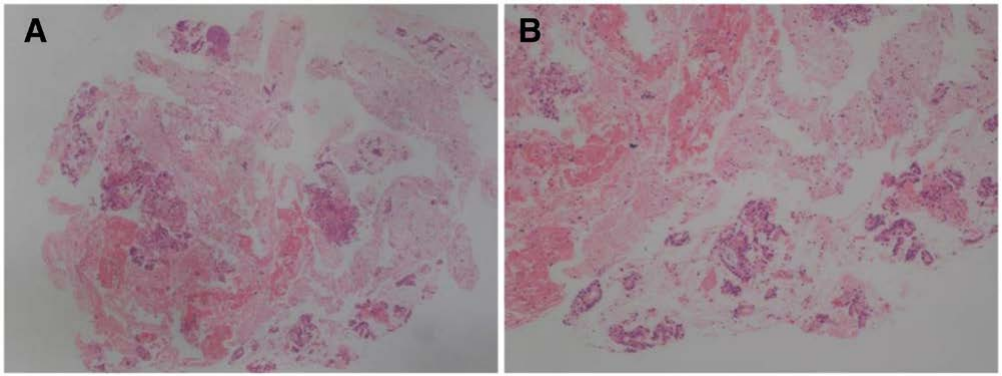
Pathological findings on hematoxylin-eosin stained sections. (A) (40×); (B) (100×).

Endoscopic ultrasound-guided fine-needle aspiration was then performed, and pathological examination indicated pancreatic adenocarcinoma. Liver puncture and microwave ablation of the liver lesion were performed. Liver puncture and pathological examination showed adenocarcinoma (Fig. 4A and B), and immunohistochemistry showed MUC1 (+), CK7 (+), SMAD4 (+), TTF-1 (−), CDX-2-88 (−), CK20 (−), SATB2 (−), and NapsinA (−). The liver adenocarcinoma was considered to be metastasized from the pancreas after the medical history and immunohistochemistry were taken into account. Therefore, the woman was diagnosed with liver metastases of pancreatic cancer during lung cancer immunotherapy. Combination of chemotherapy (gemcitabine and nba-paclitaxel) was subsequently used as the first-line chemotherapy while the anti-PD-1 immunotherapy continued.

Reexaminations showed that the pulmonary lesion was stable, but the metastatic tumor of liver continued to grow, and the pancreatic lesion gradually increased. The chemotherapy was changed to oral intake of tegafur, gimeracil, and oteracil potassium, as the patient could not tolerate the venous chemotherapy, while the anti-PD-1 immunotherapy was continued according to the schedule.

Her condition suddenly worsened at the end of 2021, and she did not come to the hospital for further treatment. The patient died on March 2022.

### 2.2. Case 2

A 69-year-old man was diagnosed with a space-occupying lesion in the kidney in February 2020. He had no history of diseases and no family history of cancer. CT scanning suggested malignancy in the right kidney accompanied by the enlargement of multiple renal hilar and retroperitoneal lymph nodes. Unilateral nephrectomy + retroperitoneal lymph node dissection was performed on February 19, 2020. Pathological examination showed clear cell carcinoma of the right kidney and lymph node cancer metastasis between the abdominal aorta and inferior vena cava (3/4). Immunohistochemistry showed Vimentin (+), CD10 (+), CK7 (−), CK8 (+), CK20 (−), Ki67 positive rate 50%, CD117 (−), TFE-3 (−), SDHB (+), and AmAcR (weak +) in super A tumor cells in right kidney. The patient was then treated with oral axitinib.

Positron emission tomography (PET)-CT examination, which was performed 7 months after the operation, showed F-fluorodeoxyglucose (FDG) uptake elevation in multiple lymph nodes posterior to the right diaphragm angle and inferior vena cava.

**Figure 5.**
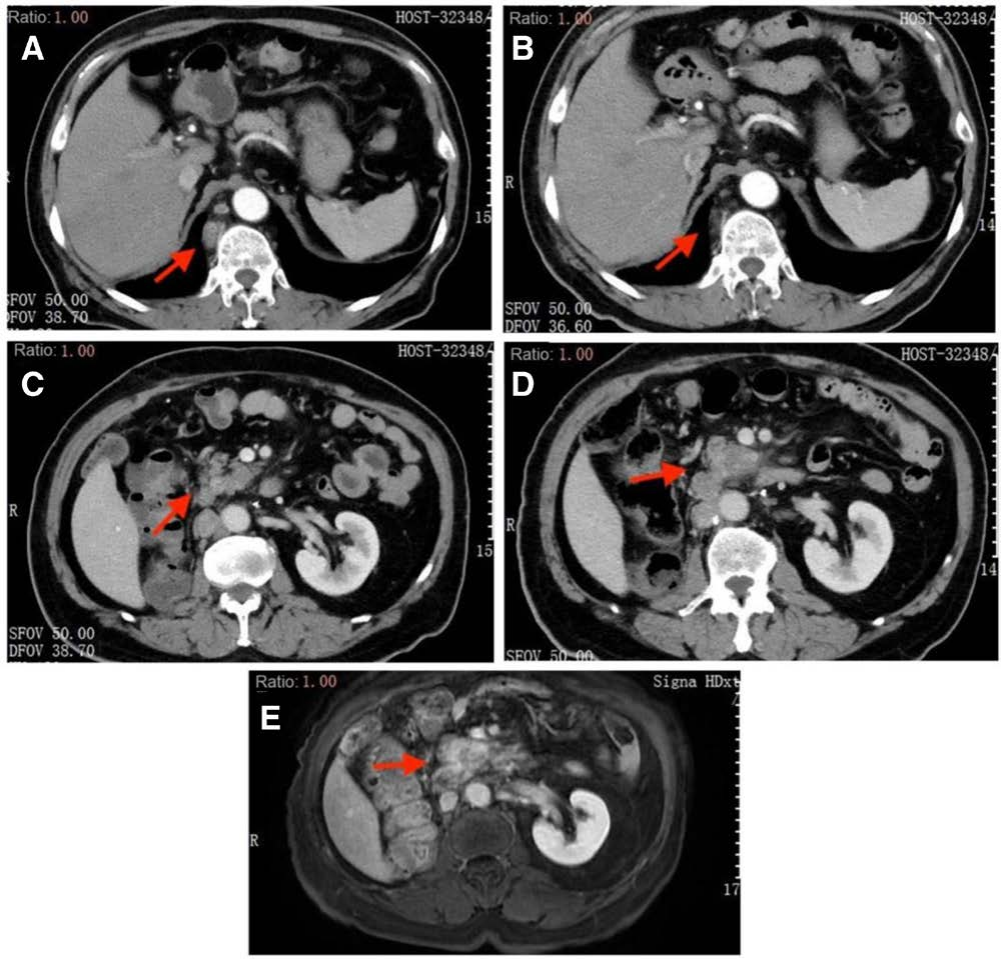
CT scanning images of the patient. The red arrow showed the major lesion. (A) CT scanning 7 months after radical therapy of renal carcinoma on December 15, 2020 showed enlargement of lymph nodes posterior to the right diaphragm angle. (B) CT scanning after 4 cycles of combination therapy by immunotherapy and targeted-therapy on March 30, 2021, showed substantial shrinking of the lymph nodes posterior to the right diaphragm angle. (C) Abdominal CT scanning on December 15, 2020, showed no abnormality in the pancreas. (D) CT scanning after 4 cycles of combination therapy by immunotherapy and targeted-therapy on March 30, 2021, showed substantial shrinking of lymph nodes posterior to the right diaphragm angle, but a low-density lesion appeared at the uncinate process of the pancreas. (E) Abdominal MRI examination showed the malignancy at the uncinate process of the pancreas. CT = computed tomography.

On December 15, 2020, the reexamination showed partial enlargement of multiple lymph nodes posterior to the right diaphragm angle and inferior vena cava (Fig. 5A and C). The patient was diagnosed with metastatic clear cell renal carcinoma (mccRCC)m and was given anti-PD-1 plus targeted-therapy (axitinib plus immunotherapy by PD-1 inhibitor) from January 6, 2021.

**Figure 6.**
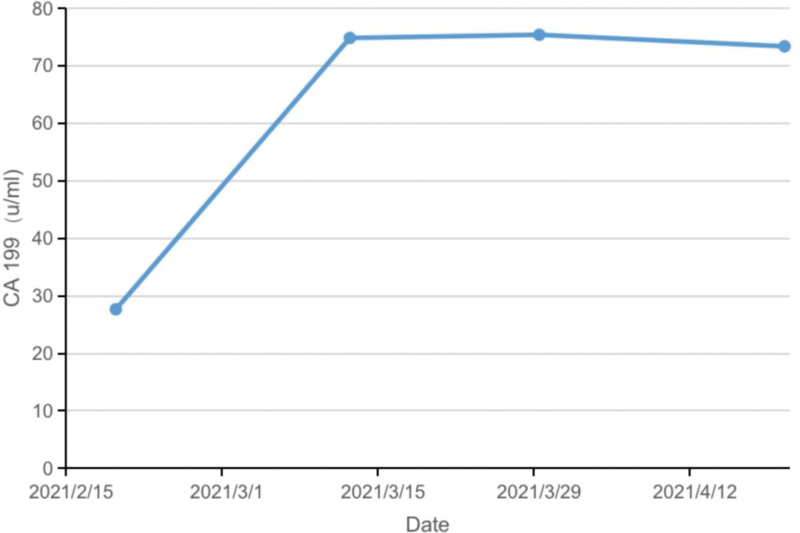
Changes of CA19-9 from February 19, 2021 to April 20, 2021.

Reexamination by CT scanning was performed after 4 cycles of the combination therapy by immunotherapy and targeted-therapy (March 30, 2021), which showed that the multiple lymph nodes posterior to the right diaphragm angle and inferior vena cava shrank compared to that observed on December 15, 2020 (Fig. 5B), and a low-density lesion at the uncinate process of the pancreas (Fig. 5D). The assessment showed that the treatment efficacy was PR. However, a new lesion appeared in patient’s pancreas. Magnetic resonance imaging (MRI) scanning was performed, showing malignancy at the uncinated process of the pancreas (Fig. 5E). CA19-9 examination also showed that the level increased from 27.66 to 73.36 µ/mL (Fig. 6).

**Figure 7.**
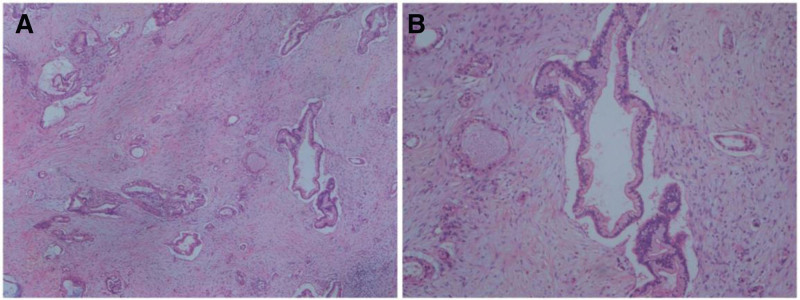
Pathological findings on hematoxylin-eosin stained sections. (A) (40×); (B) (100×).

Endoscopic ultrasound-guided fine-needle aspiration was performed for pancreas puncture and biopsy on May 8, 2021, and pathological examination suggested pancreatic cancer. The patient was diagnosed with a respectable pancreatic head tumor. Gastroduodenal pancreatectomy was performed on August 12, 2021, and postoperative pathological examination showed highly differentiated pancreatic cancer at the pancreatic head duct (Fig. 7A and B), which invaded the adipose tissues, lymph nodes, nerves, and vessels surrounding the pancreas. Immunohistochemistry showed EGFR (+/−), P53 (weak +), HER2 (0), E-Cad (+), MLH1 (ES05) (+), MSH2 (+), MSH6 (partial +), PMS2 (+), P16 (−), SMAD4 (−/+), C-myc (partial +), and ki67 + CD8 (+5%, partial +). Finally, the man was diagnosed with second primary malignant pancreatic head carcinoma. The patient refused chemotherapy, while the treatment with Axitinib plus PD-1 inhibitor was continued. The last follow-up of the patient was on January 5, 2022, and the reexamination showed the disease was stable. The anti-PD-1 therapy in the patient was 12 months in total, and the overall survival was more than 1 year. Although the immunotherapy persistently controlled the metastases in lymph nodes, the second primary malignancy appeared during the treatment, which was fortunately detected and treated by surgical resection. The major adverse reaction during the combination therapy by immunotherapy and targeted-therapy was diarrhea.

## 3. Discussion

Multiple primary malignant neoplasm (MPMN) refers to 2 or more histologically different tumors simultaneously occurring in the same patient. The tumors could be restricted to 1 organ or various anatomical organs.^[3]^ To the best of our knowledge, these 2 cases are the first reports of the second primary malignancy that occurred during immunotherapy, and the lesions in both patients were malignant pancreatic tumors. These findings indicated that with the extensive application of immunotherapy and the progression of diagnosis and treatment regimens, the disease course of malignancy and the risk of MPMN occurrence both increased.

The first case reported here was a patient with stage III NSCLC. According to the Asian Thoracic Oncology Research Group Expert Consensus Statement on combination management, an multidisciplinary discussion team discussion was performed, suggesting that surgery or radiotherapy was unsuitable for the patient. The emergence of ICI has completely changed the treatments for metastatic/recurrent patients, and the introduction of consolidation durvalumab in the treatment of unresectable stage III NSCLC has also led to the change in treatment regimens for stage III NSCLC.^[4]^ This patient was treated with chemotherapy, and anti-PD-1 treatment was performed throughout the consequent treatments. The pulmonary lesion in the patient did not progress (18 months), and the adverse reactions were tolerable, which further demonstrated that patients with unresectable stage III NSCLC could benefit from immunotherapy. Unfortunately, primary pancreatic malignancy appeared during the treatment, and liver metastases were detected. The findings in the consequent treatment showed that the immunotherapy was noneffective for the second primary malignancy. The primary malignancy in the lung was well controlled, but the second primary malignancy still progressed continuously.

The second case reported here was a patient with mccRCC. The combination therapy by ICI and tyrosine kinase inhibitor was selected as the new standardized treatment for the mccRCC patient according to the CLEAR test results.^[2]^ The findings of a randomized phase 3 KEYNOTE-426 trial^[5]^ showed that mortality risk was 47% lower in the pembrolizumab-axitinib group than in the sunitinib group. Moreover, the JAVELIN Renal 100 study^[6]^ (avelumab plus axitinib as first-line therapy in patients with advanced clear cell renal-cell carcinoma) also showed high antitumor activity and safety of the treatment. Our patient was treated by ICI and VEGF pathway inhibitor, which achieved disease remission (substantial shrinkage of retroperitoneal lymph nodes), and the disease did not progress till the follow-up. Unfortunately, the second primary pancreatic malignancy occurred during the immunotherapy, but the patient was more fortunate than the first patient, as the pancreatic malignancy was resectable, and the patient was treated by surgical resection in time. The patient had been treated with anti-PD-1 for 12 months in total till the follow-up, and the overall survival was >1 year.

The findings in these 2 cases demonstrated the sensitivity of CA19-9. A previous study on the serum of patients with prostate, lung, colorectal, and ovarian cancers from the Prostate, Lung, Colorectal and Ovarian (PLCO) (Cancer Screening Trial of the National Cancer Institute) cohort has already documented the effect of CA19-9 in detecting pancreatic cancer in asymptomatic subjects.^[7]^ The elevation of CA19-9 appears 2 years earlier than the clinical diagnosis of the disease. More importantly, the detection of CA19-9 could also provide an important time window for the detection of resectable diseases. Patients with primary malignancies have a higher risk of developing second primary cancer.^[8]^ Therefore, active screening of the second primary malignancy is necessary during or after the treatments for the first primary malignancy. Furthermore, the following issues need to be considered during the treatment: the efficacy of ICIs in different types of tumors and different individuals varies substantially. The anti-PD-1 therapy was effective in the first case but had no clinical effect on the pancreatic malignancy. Identifying reliable biomarkers for predicting the clinical response to immunotherapy in different malignancies has become an urgent requirement; several studies have demonstrated that chemotherapy and radiotherapy could elevate the incidence of second primary malignancy.^[9,10]^ However, the relationships of immunotherapy with long-term and short-term second primary malignancy incidence are still unclear and require further long-term investigation. Regarding the treatment, we suggested NGS to both patients, but neither accepted the suggestion. NGS is necessary to clarify the common originations and mutation genes in malignancies. In immunotherapy, no standard guideline for treating MPMN is available. However, the type of malignancy, disease course, response to treatment, and general patient characteristics should all be considered. If the diseases are curable, radical treatment should be selected by the doctors. Immunotherapy was continued for the 2 patients as the primary lesion was still in persistent remission. However, local treatment such as surgery, radiotherapy, ablation, and interventions should also be considered for the second primary malignancies. The relationships and influences between the local treatments and immunotherapy, positive or negative, need to be further investigated in clinical practice.

## 4. Conclusion

The first case was treated by chemotherapy plus immunotherapy and consequent maintenance immunotherapy, and the second case was treated by targeted-therapy plus immunotherapy. Both patients achieved disease remission, and chemotherapy, targeted-therapy, and immunotherapy achieved synergistic effects. However, second primary malignancy occurred in both patients during the immunotherapy, and more clinical studies and practices are still needed for the adjustment of the regimens of immunotherapy. In addition, we noticed that during the treatment of malignancies in patients, the second tumor needs to be screened. Full diagnosis, timely treatment, and long-term regular follow-up have important significance for the patients with malignancies.

## Author contributions

**Conceptualization:** Xian Miao.

**Data curation:** Xian Miao.

**Formal analysis:** Xiaohui Yang.

**Funding acquisition:** Shuijie Shen.

**Investigation:** Yuhua Tao.

**Methodology:** Shu Dong.

**Project administration:** Shuijie Shen.

**Resources:** Shuijie Shen.

**Software:** Shu Dong.

**Supervision:** Shuijie Shen.

**Validation:** Shu Dong.

**Visualization:** Xiaohui Yang.

**Writing—original draft:** Xian Miao.

**Writing—review & editing:** Xian Miao.
